# Cost-Effectiveness of Radiotherapy and Its Impact on Patient Quality of Life: A Real-World Cost Utility Analysis in Greece

**DOI:** 10.3390/curroncol33040220

**Published:** 2026-04-16

**Authors:** Elissavet Vardaki, Maria Tolia, Christos Michalakelis, Athanassios Vozikis

**Affiliations:** 1 Medical School, University of Crete, Panepistimiou 1, 71100 Herakleio, Greece; mariatolia@uoc.gr; 2 Department of Radiation Oncology, University Hospital of Heraklion, 71110 Crete, Greece; 3 Department of Informatics and Telematics, Harokopio University of Athens, 17778 Tavros, Greece; michalak@hua.gr; 4 Laboratory of Health Economics and Management, Department of Economics, University of Piraeus, 18534 Piraeus, Greece; avozik@unipi.gr

**Keywords:** cancer, radiotherapy, cost-effectiveness analysis, QALY, financial toxicity

## Abstract

Background: The aim of this study was to estimate the economic burden of radiotherapy (RT) from the perspectives of payers, the healthcare system, patients, and society, and to assess associated quality-of-life (QoL) outcomes. The analysis examined direct medical and non-medical costs, as well as QoL, before, during, and up to six months after RT. Given the inclusion of multiple cancer types, the study reflects a heterogeneous real-world population. An exploratory comparison across RT techniques was also conducted to provide contextual economic insight. Methods: This analysis included data from 301 cancer patients undergoing RT using various techniques, including two-dimensional radiotherapy (2D), 3D conformal radiotherapy (3D-CRT), volumetric-modulated arc therapy (VMAT), and intensity-modulated radiotherapy (IMRT), at the University General Hospital of Heraklion, Crete, Greece. Clinical and cost data were collected retrospectively, while QoL data were collected prospectively using validated instruments at baseline, end of treatment, and six months post-treatment. Quality-adjusted life years (QALYs) were estimated. The primary analysis compared RT with a hypothetical “no RT” comparator derived from published evidence, while comparisons across RT techniques were conducted as exploratory analyses. Costs and QALYs were evaluated over a 6-month time horizon; therefore, discounting was not applied. Incremental cost-effectiveness ratios (ICERs) were calculated, and probabilistic sensitivity analysis was performed to account for parameter uncertainty. Results: The cost per QALY gained with RT compared with the hypothetical “no RT” comparator varied substantially across techniques and cancer types. In the primary analysis, 2D radiotherapy yielded the lowest ICER (€13,043.27/QALY), while VMAT demonstrated an ICER of €29,945.12/QALY. In contrast, IMRT was associated with a substantially higher ICER (€135,529.51/QALY), suggesting limited cost-effectiveness under commonly accepted willingness-to-pay thresholds, whereas 3D-CRT was found to be dominant. Subgroup analyses revealed marked heterogeneity, with ICERs ranging from €3234.45 to €30,232.50 per QALY gained across cancer types. In certain subgroups, RT was either cost-saving or dominant, particularly in breast cancer (cost-saving with similar QALYs) and in skin cancer and sarcoma (dominant strategies). Sensitivity analyses highlighted considerable uncertainty, especially for 2D radiotherapy, primarily driven by small sample sizes and variability in QALY estimates. Conclusions: This study provides short-term, real-world evidence on the cost-effectiveness and quality-of-life outcomes of radiotherapy in a Greek healthcare setting. While simpler techniques such as 2D radiotherapy may appear economically favorable, their limited effectiveness and substantial uncertainty may reduce their overall value. In contrast, advanced techniques—particularly VMAT—demonstrate a more consistent balance between cost and clinical outcomes, supporting their role within value-based, patient-centered oncology care. However, the findings should be interpreted with caution due to population heterogeneity, small subgroup sizes, the short (6-month) time horizon, and the use of a hypothetical comparator. Further research with longer follow-up and disease-specific analyses is warranted.

## Simple Summary

Radiotherapy is a key component of cancer treatment, but its economic impact and effects on patients’ quality of life remain underexplored in real-world settings, particularly in Greece. This study examined the short-term costs and quality-of-life outcomes of different radiotherapy techniques using patient-level data from a Greek hospital. The findings indicated that more advanced techniques, such as IMRT and VMAT, were associated with improved quality of life, although costs and outcomes varied across cancer types. Due to differences in patient characteristics, disease types, and the short follow-up period, the results should be interpreted with caution. Overall, this study provides real-world evidence that may support future research and healthcare decision-making in oncology.

## 1. Introduction

Modern radiotherapy (RT) techniques, such as intensity-modulated radiotherapy (IMRT) and volumetric-modulated arc therapy (VMAT), have improved dose distributions, reduced exposure to surrounding healthy tissues, and enhanced clinical outcomes. Previous studies suggest that these techniques may be associated with improved quality of life (QoL) and potentially better economic outcomes in selected malignancies [1,2]. IMRT has demonstrated improved organ sparing and patient-reported outcomes, especially in head and neck cancers [3], while VMAT offers comparable or better outcomes with shorter treatment times [4].

Economic evaluations in oncology often assess the balance between higher upfront costs and potential gains in quality-adjusted life years (QALYs), using metrics such as incremental cost-effectiveness ratios (ICERs) and, where appropriate, willingness-to-pay (WTP) thresholds [5,6]. However, the interpretation of such results may vary depending on factors such as methodological assumptions, data availability, and context-specific factors, particularly in resource-constrained healthcare systems.

In the Greek setting, there is limited evidence based on real-world patient-level data, and no national EQ-5D-5L value set is currently available [7]. However, despite the growing use of advanced radiotherapy techniques, there is limited real-world evidence integrating both cost and quality-of-life outcomes, particularly in the Greek healthcare setting. Therefore, this study aims to assess the cost and quality-of-life outcomes associated with different RT techniques (2D, 3D-CRT, IMRT, and VMAT) in a cohort of Greek cancer patients, incorporating real-world cost and QoL data, rather than to establish definitive cost-effectiveness conclusions or demonstrate the superiority of one RT technique over another. Given the limited follow-up period, the present analysis focuses on short-term cost and quality-of-life outcomes. In this context, comparisons with a “no RT” scenario should be interpreted as exploratory and with caution due to underlying assumptions. Additionally, a probabilistic sensitivity analysis was applied to assess the robustness of the findings under parameter uncertainty.

## 2. Materials and Methods

### 2.1. Methodology

#### 2.1.1. Population and Study Design

The present study used a mixed design, with retrospective collection of clinical and cost data and prospective collection of quality-of-life data, using real-world data from 301 cancer patients who underwent RT at the University Hospital of Heraklion, Crete, Greece. The cohort included patients with a range of cancer types treated with various RT techniques (2D, 3D-CRT, Intensity Modulated Radiotherapy (IMRT), and Volumetric Modulated Arc Therapy (VMAT)), reflecting clinical heterogeneity. Data collection focused on clinical characteristics, RT techniques, and economic data, specifically direct medical and non-medical costs.

This economic evaluation was conducted primarily from a healthcare system perspective, while direct non-medical costs were additionally included to capture elements of a broader societal perspective. The time horizon covered the treatment period and extended to six months following radiotherapy completion to capture short-term outcomes and quality-of-life changes [5,6]. Discounting was not applied due to the short (6-month) time horizon.

#### 2.1.2. Measurement of Quality of Life and QALYs

Quality of life (QoL) was assessed using both generic and disease-specific instruments. The EQ-5D-5L questionnaire was used to estimate health-related quality of life (HRQoL) and to derive utility scores, which were subsequently converted into quality-adjusted life years (QALYs) using the area under the curve (AUC) method [8,9,10]. As no national EQ-5D-5L tariff currently exists in Greece, the Spanish value set was adopted, in line with other European health economic evaluations [11]. The cancer-specific EORTC QLQ-C30 was also administered, and together with EQ-5D-5L, it provided a more nuanced understanding of patients’ health-related quality of life during radiotherapy [7].

## 3. Cost Assessment

### 3.1. Direct Medical Costs

The economic evaluation was conducted from a healthcare system perspective. Direct medical costs included expenditures related to radiotherapy procedures, hospital services, and other healthcare resource utilization. These costs were derived from National Health Service Organization (EOPYY) invoices [12], the Greek Diagnosis-Related Group (DRG) system (Closed Unified Hospital Charges—KEN) [13], and actual hospital cost data. This approach ensures alignment with national reimbursement mechanisms and reflects real-world cost structures within the Greek healthcare system, as recommended in health economic evaluations [8].

### 3.2. Direct Non-Medical Costs

Direct non-medical costs included transportation, patient accompaniment, and other out-of-pocket expenses related to cancer treatment.

This approach ensures alignment with national reimbursement mechanisms and reflects real-world cost structures within the Greek healthcare system, as recommended in health economic evaluations [8].

## 4. Calculation of ICER

The incremental cost-effectiveness ratio (ICER) was calculated as the difference in costs divided by the difference in quality-adjusted life years (QALYs) between radiotherapy and the “no RT” comparator (ICER = ΔCosts/ΔQALYs).

Because no real-world comparator group of patients not receiving radiotherapy was available in the present study, a hypothetical “no RT” comparator was constructed using cost and utility estimates derived from published economic evaluations of palliative and best supportive care pathways. Specifically, studies such as Kramer et al. (2024) [14] and Mulvenna et al. (2016) [15] were used to approximate costs and outcomes associated with alternative care pathways without radiotherapy. This approach introduces additional uncertainty and limits direct comparability with real-world untreated populations.

Although these studies did not always explicitly isolate a “no RT” arm, subgroup data and reported outcomes were used to derive proxy estimates for comparative purposes. Therefore, the ICER estimates based on the hypothetical “no RT” comparator should be interpreted with caution due to the indirect nature of the comparator, while comparisons across RT techniques are considered exploratory.

A commonly used willingness-to-pay (WTP) threshold of €30,000 per QALY gained was applied to interpret cost-effectiveness results, in line with thresholds applied in European health economic evaluations [8,16].

## 5. Sensitivity Analysis

A probabilistic sensitivity analysis (PSA) was performed using Monte Carlo simulations with 10,000 iterations to address uncertainty in model inputs, in accordance with internationally accepted health technology assessment (HTA) guidelines [6,17].

Gamma distributions were assigned to cost parameters, as costs are typically continuous, positively skewed, and restricted to non-negative values. Beta distributions were applied to utility values (used to estimates QALYs), as these are bounded between 0 and 1 and are appropriate for modeling health-related quality of life [17].

The parameters of the Gamma distribution (k and θ) were calculated as follows:

k = (mean/SD)^2^

θ = SD^2^/mean

For the Beta distribution (α and β), the following formulas were used:

α = mean × [(mean × (1 − mean)/variance) − 1]

β = (1 − mean) × [(mean × (1 − mean)/variance) − 1]

When calculated Beta parameters resulted in invalid values (e.g., negative shape parameters), the standard deviations were adjusted, and the parameters were recalculated to ensure valid and stable Beta distributions.

The results of the PSA were used to generate cost-effectiveness acceptability curves (CEACs), to estimate the probability of cost-effectiveness across a range of WTP thresholds (€20,000–€50,000 per QALY gained) [16].

## 6. Quality of Life Analysis

Patients receiving radiotherapy (RT) were assessed using the EORTC QLQ-C30 questionnaire three times: before treatment, at the end of treatment, and at 6-month follow-up. These instruments enabled the assessment of both disease-specific and generic health-related quality of life (HRQoL) [12].

Quality-adjusted life years (QALYs) were derived from EQ-5D-5L data using the Spanish value set, due to the absence of a national Greek tariff [7].

Quality-of-life outcomes were evaluated across the different RT techniques included in the study. Four RT techniques were analyzed: two-dimensional radiotherapy (2D), three-dimensional conformal radiotherapy (3D-CRT), intensity-modulated radiotherapy (IMRT), and volumetric-modulated arc therapy (VMAT) [1,2,3,4].

## 7. Results

### 7.1. Patient Characteristics

The final sample included 301 oncology patients treated with RT between 2021 and 2022 at the University Hospital of Heraklion. The distribution by RT technique was as follows: 3D-CRT (18.6%), IMRT (50.5%), VMAT (29.2%), and a small proportion treated with 2D (1.7%). Cancer diagnoses covered multiple cancer types. The mean age was 62.5 years, with 58.1% females and 41.9% males. Of the total costs, 80% were direct medical costs and 20% were direct non-medical costs.

### 7.2. Costs and QALYs by Treatment Strategy

The mean costs and QALYs per patient are summarized in Table 1. A hypothetical No RT comparator was constructed using literature-derived estimates for cost and QALYs in palliative or best supportive care settings. This approach is commonly employed in cost-effectiveness analyses when randomized controlled data are unavailable.

As shown in Table 1, 3D-CRT was found to be dominant compared with no RT, as it was associated with lower costs and slightly higher QALY values. In contrast, 2D and IMRT were associated with lower costs but also lower QALY values, indicating lower effectiveness compared with no RT. VMAT was associated with higher costs and higher QALY values, resulting in an ICER of €29,945.12 per QALY.

These findings are consistent with the previous literature on the economic evaluation of radiotherapy techniques, which suggests that advanced modalities may improve clinical outcomes and quality of life, albeit at increased costs [18,19]. The ICER values observed for IMRT (€135,529.51/QALY) and VMAT (€29,945.12/QALY) suggest that VMAT may be considered cost-effective under commonly accepted willingness-to-pay thresholds [16,20], whereas IMRT is unlikely to be cost-effective.

However, these findings should be interpreted with caution given the use of a hypothetical comparator. Overall, while some conventional techniques such as 2D may appear less costly but less effective, 3D-CRT demonstrated a dominant profile, whereas advanced radiotherapy techniques—particularly VMAT—may offer greater value in terms of cost-effectiveness due to improved patient outcomes.

### 7.3. Cost-Effectiveness by Cancer Type

Subgroup analysis by cancer type revealed considerable variability in cost-effectiveness outcomes. ICER values for each cancer type were estimated using the “No RT” comparator, based on literature-derived cost and utility estimates, as described in the methodological framework of the study. Specifically, studies such as Mulvenna et al. (2016) [15], which included supportive-care-only arms, were used to inform model inputs for constructing non-RT care pathways. Where clearly isolated non-RT arms were unavailable, QALY and cost inputs were reconstructed from subgroup data and graphical outputs, following established methodological guidance for secondary data extraction [21,22]. Details of the reconstruction process and derived input parameters are provided in Table S1. Cancer-specific ICER results are presented in Table 2.

The results showed variability across cancer types, reflecting the heterogeneity of the included cancer populations and representing a limitation of the analysis, as pooling cancers with different prognoses and treatment intents may reduce clinical interpretability. Radiotherapy was found to be dominant in skin cancer and sarcoma, indicating lower costs and higher QALYs compared with no RT. This dominance is primarily driven by lower treatment-related costs and reduced healthcare resource utilization associated with radiotherapy compared with the “no RT” comparator. In breast cancer, radiotherapy was cost-saving with equal QALYs.

Across the remaining cancer types, ICER estimates indicated a mix of outcomes, including greater effectiveness at higher costs, cost savings accompanied by reduced effectiveness, and in some cases, dominant strategies. ICER values ranged from €3234.45 to €30,232.50 per QALY gained, although estimates associated with negative QALY differences should be interpreted with caution. Lower ICERs were observed in lung cancer (€13,170.19/QALY), while higher values were identified in bladder (€30,232.50/QALY) and endometrial cancer (€29,621.33/QALY).

Head and neck cancer showed an ICER of €22,275.00 per QALY, while prostate and rectal cancers had ICERs of €23,160.00 per QALY and €22,722.86 per QALY, respectively. Pancreatic and stomach cancers also demonstrated moderate ICER values (€10,805.56/QALY and €23,404.13/QALY, respectively).

The negative QALY values observed in some subgroups (e.g., bone metastasis) reflect poor health states associated with advanced disease and should be interpreted with caution, particularly when evaluating cost-effectiveness estimates.

Overall, many ICER estimates fell below commonly accepted willingness-to-pay thresholds, suggesting that radiotherapy may represent a cost-effective option for several cancer types. However, these findings should be interpreted cautiously given the exploratory nature of the analysis and the use of a hypothetical “no radiotherapy” comparator constructed from literature-derived estimates, which introduces additional uncertainty and limits direct comparability with real-world untreated populations [16,20].

### 7.4. Probabilistic Sensitivity Analysis (PSA)

A Probabilistic Sensitivity Analysis (PSA) was conducted using 10,000 Monte Carlo simulations to account for uncertainty in cost and QALY estimates across treatment strategies. Cost parameters were modeled using Gamma distributions due to their skewed, non-negative nature, while QALYs were modeled using Beta distributions, as they are bounded between 0 and 1. Input parameters for each distribution, including means and standard deviations, were derived from primary patient-level data and supplemented by literature-based estimates for the comparator group (best supportive care). Corresponding distribution parameters were calculated to appropriately reflect uncertainty in the probabilistic model [17,22].

Table 3 presents the estimated distribution parameters for each radiotherapy technique and the comparator group. VMAT and 3D-CRT demonstrated higher mean QALY values compared with other techniques, while their Beta distributions were more concentrated, indicating reduced variability. In contrast, 2D radiotherapy exhibited greater dispersion in QALY estimates (α = 0.0325; β = 0.2028), suggesting higher uncertainty in effectiveness outcomes.

Similarly, cost uncertainty was highest in the VMAT group, as indicated by the wider Gamma distribution (k = 14.98; θ = 602.09). These probabilistic inputs were subsequently used to generate cost-effectiveness acceptability curves (CEACs) and cost-effectiveness planes, which illustrate the probability that each strategy is cost-effective across a range of willingness-to-pay thresholds. The results showed that VMAT had the highest probability of being cost-effective across a wide range of willingness-to-pay thresholds. These findings reflect the underlying uncertainty in model inputs, particularly in cost and QALY estimates, and highlight the importance of probabilistic analysis in supporting decision-making.

### 7.5. Deterministic Sensitivity Analysis (DSA)

A deterministic sensitivity analysis (DSA) was conducted to evaluate the impact of uncertainty in key BSC parameters on the model outcomes. Cost and QALY inputs for the comparator parameters were varied by ±20%, and the corresponding ICER values were recalculated. The ICER ranged from 23,092.46 to 57,090.19 €/QALY when varying the cost of the comparator, and from 7040.70 to 9351.36 €/QALY when varying QALYs. Overall, the model was more sensitive to variations in cost parameters than QALY estimates, while ICER estimates remained within a plausible range (Table 4).

### 7.6. Cost-Effectiveness Acceptability Curve (CEAC)

The Cost-Effectiveness Acceptability Curve (CEAC) illustrates the probability that each RT technique is cost-effective across the full range of WTP thresholds, derived from the probabilistic sensitivity analysis. Among the evaluated modalities, 2D RT appeared to have an approximately a 90% probability of being cost-effective at a WTP threshold of €30,000/QALY and maintained relatively high probabilities across the entire WTP range. However, the 2D group had the smallest sample size (*n* = 5), which reduces the reliability of its probabilistic estimates. Moreover, its lower average QALY gain (0.1394) compared with 3D-CRT (0.5230), IMRT (0.3820), and VMAT (0.4956) indicates substantially limited health benefits. Thus, the apparently favorable ICER for 2D suggests that its apparent cost-effectiveness may be driven primarily by lower cost rather than substantial health gains.

VMAT showed an approximate 50% probability of cost-effectiveness, while IMRT and 3D-CRT demonstrated lower probabilities of cost-effectiveness at higher WTP levels. These differences reflect both variation in QALY gains and differing degrees of cost uncertainty among the techniques. The CEAC provides a clear visual summary of the probability that each RT modality remains cost-effective under uncertainty and is widely used to support reimbursement, guideline development, and clinical adoption decisions, particularly in resource-constrained settings [16,17,22]. The CEAC graph of all RT techniques is presented in Supplementary Figure S1. Overall, no single strategy was consistently dominated across all WTP thresholds.

### 7.7. Cost-Effectiveness Plane (CE Plane)

The cost-effectiveness plane (CE plane) provides a graphical visualization of the joint uncertainty in incremental costs and QALYs for each RT technique based on 10,000 Monte Carlo simulations. Most simulations for the 2D technique fall predominantly in the south-west quadrant, indicating lower costs but also lower QALYs relative to the comparator. In contrast, VMAT and IMRT techniques cluster predominantly in the north-east quadrant, indicating higher costs and greater effectiveness. Overall, the aggregated results suggest that the 2D RT technique is associated with lower costs but also lower QALYs compared with no-RT comparator, whereas IMRT and VMAT demonstrated higher effectiveness at higher costs.

To supplement ICER-based analysis and address its limitations under uncertainty, the Net Monetary Benefit (NMB) approach was applied across willingness-to-pay (WTP) thresholds ranging from €0 to €60,000/QALY. The NMB framework enables direct comparison of treatments by transforming costs and effects into a single monetary metric, thereby facilitating decision-making under probabilistic frameworks [17,22]. NMB is defined as:

NMB = (QALY × WTP) − Cost

Across the full range of WTP thresholds, the 2D technique demonstrated higher probability of cost-effectiveness at lower thresholds (e.g., 94.5% at €0, 82.6% at €5000), primarily driven by its lower cost profile rather than substantial improvements in effectiveness. However, this result should be interpreted cautiously due to the small sample size (*n* = 5), which may limit the robustness and generalizability of these findings, particularly given its lower average QALY gain compared to other techniques. As WTP increases, IMRT and VMAT become increasingly favorable, with VMAT exceeding a 50% probability of cost-effectiveness at thresholds above €40,000/QALY [16]. This likely reflects greater effectiveness, particularly in reducing toxicity and preserving quality of life, as demonstrated in published evaluations of modern conformal radiotherapy techniques [3,4].

These findings support the utility of the NMB framework in assessing RT cost-effectiveness under uncertainty and complement the insights obtained from CEAC and ICER analyses [23,24]. The CE plane scatter plot is shown in Figure S2, the CE plane results are presented in Figure S3, and the NMB values for each WTP threshold and technique are reported in Table S2.

### 7.8. Quality of Life Analysis and Comparative Assessment of Radiotherapy Techniques

Multivariate analysis of variance (MANOVA) revealed statistically significant effects of RT technique on several EORTC QLQ-C30 domains, including global health status (GHS), functional scales, and symptom burden across three time points: baseline, end of treatment, and 6-month follow-up.

At baseline, the effect of RT technique was significant for GHS and baseline quality-of-life scores (Wilks’ λ = 0.895, F(6, 592) = 5.61, *p* < 0.001, partial η^2^ = 0.054). At the end of treatment, RT technique had a stronger impact on functional outcomes and symptom burden (Wilks’ λ = 0.865, F(24, 876) = 2.04, *p* = 0.003, partial η^2^ = 0.047), with further significant effects at the 6-month follow-up (Wilks’ λ = 0.851, F(24, 864) = 2.04, *p* < 0.001, partial η^2^ = 0.054).

Regarding functional domains, patients treated with IMRT and VMAT demonstrated significantly better outcomes in physical, role, emotional, and social functioning at follow-up compared with those treated with 2D or 3D-CRT, with VMAT consistently achieving the highest functional scores over time. Statistically significant differences between techniques were observed for physical functioning (F(3, 297) = 6.41, *p* < 0.001), role functioning (F(3, 297) = 6.75, *p* < 0.001), and social functioning (F(3, 297) = 6.45, *p* < 0.001), with partial η^2^ values ranging from 0.061 to 0.076, indicating moderate effect sizes. These findings suggest improved functional recovery with modern conformal techniques.

In terms of symptoms, patients treated IMRT or VMAT experienced lower symptom burden, particularly in fatigue, pain, and nausea/vomiting. Statistically significant differences were observed across multiple domains, including fatigue (F(3, 297) = 6.41, *p* < 0.001), pain (F(3, 297) = 8.27, *p* < 0.001), nausea/vomiting (F(3, 297) = 8.26, *p* < 0.001), insomnia (F(3, 297) = 10.52, *p* < 0.001), appetite loss (F(3, 297) = 8.02, *p* < 0.05), constipation (F(3,297) = 1.97, *p* < 0.05), diarrhea (F(3, 297) = 4.64, *p* = 0.003), and dyspnea (F(3, 297) = 8.25, *p* < 0.001).

Collectively, VMAT demonstrated the most favorable overall profile, followed by IMRT. This pattern suggests reduced toxicity and symptom burden with modern RT techniques compared with conventional approaches.

Additionally, global health status (GHS) improved significantly across techniques. Post hoc pairwise comparisons with Bonferroni correction indicated that VMAT and IMRT yielded higher GHS scores at the end of treatment and at 6 months post-treatment (F(3,297) = 5.77, *p* < 0.001, partial η^2^ = 0.055). Baseline QLQ-C30 scores were also significantly associated with technique (F(3,297) = 9.61, *p* < 0.001, partial η^2^ = 0.089), suggesting an association between technique and baseline health perception.

Overall, patients treated with modern techniques (IMRT and VMAT) demonstrated consistently better functional outcomes, reduced symptom burden, and improved global health status across all evaluated time points compared with conventional techniques (2D and 3D-CRT).

## 8. Discussion

This study investigated the comparative cost-effectiveness and quality-of-life outcomes of different radiotherapy (RT) techniques in oncology patients treated in a real-world hospital setting [25]. By integrating economic evaluation with patient-reported outcomes, the analysis provides a comprehensive assessment of the value delivered by each modality.

The primary cost-effectiveness analysis was based on a comparison between radiotherapy (RT) and a hypothetical “no RT” comparator [26], whereas comparisons across RT techniques should be interpreted as exploratory analyses.

In exploratory comparisons across RT techniques, 2D RT yielded the lowest ICER, suggesting a potentially favorable profile under strict budget constraints. However, this finding should be interpreted with caution, as the very small sample size (*n* = 5) substantially limits the robustness and generalizability of these estimates. In addition, 2D RT demonstrated the lowest QALY gains and the most limited health benefit, indicating that its apparent economic advantage is largely driven by lower upfront costs rather than meaningful clinical benefit.

In contrast, IMRT and VMAT were associated with greater QALY gains and moderate ICER values. These findings align with established willingness-to-pay (WTP) thresholds commonly applied in European settings (approximately €20,000–€30,000/QALY), supporting the interpretation that modern RT techniques may represent cost-effective options despite their higher initial costs [16,20,27]. The potential advantage of VMAT over IMRT may depend on specific clinical settings, such as tumor site, treatment complexity, and dose distribution characteristics.

The probabilistic sensitivity analysis (PSA) further highlighted substantial uncertainty in the 2D RT estimates, largely driven by the small sample size and variability in QALY outcomes. Conversely, IMRT and VMAT showed more stable and consistent probability distributions, supporting more robust cost-effectiveness inferences. In addition, the deterministic sensitivity analysis confirmed the robustness of the model, as ICER estimates remained within plausible ranges under ±20% variations in key cost and QALY parameters, with greater sensitivity observed in cost inputs. These findings provide additional confidence in the stability of the base-case conclusions despite underlying parameter uncertainty. This is consistent with previous economic modelling studies highlighting the importance of uncertainty analysis in health economic evaluations [17,22].

The cost-effectiveness acceptability curve (CEAC) showed that 2D RT had the highest probability of being cost-effective at very low WTP thresholds; however, this probability declined as thresholds increased. In contrast, IMRT and VMAT became more favorable at moderate to higher WTP levels, reflecting their greater effectiveness. These patterns were further supported by the cost-effectiveness plane, where 2D RT clustered in the low-cost/low-benefit quadrant, while IMRT and VMAT were positioned in the higher-cost/higher-effectiveness region. Overall, the aggregated results suggest that radiotherapy may represent a dominant strategy compared with no RT, as it is associated with lower costs and improved health outcomes; however, no single RT technique consistently dominates across all willingness-to-pay thresholds.

The quality-of-life analysis based on EORTC QLQ-C30 demonstrated statistically significant differences across RT techniques. Patients treated with IMRT and VMAT consistently demonstrated better functional outcomes—particularly in physical, role, emotional, and social functioning—compared with those receiving 2D RT or 3D-CRT [28,29,30,31,32]. These findings are consistent with emerging clinical evidence suggesting improved quality of life and functional recovery with modern radiotherapy techniques.

In terms of symptom burden, IMRT and VMAT were associated with lower levels of fatigue, pain, nausea/vomiting, insomnia, appetite loss, and dyspnea [30,33]. These findings are clinically and biologically plausible, as improved conformality and dose distribution may reduce radiation exposure to surrounding healthy tissues. Supporting evidence from dosimetric and clinical studies suggests that advanced techniques such as VMAT may reduce toxicity and improve treatment tolerability compared with conventional approaches [24,26,34].

Additionally, global health status (GHS) improved significantly across the evaluated techniques, with post hoc comparisons indicating that IMRT and VMAT achieved higher scores both at treatment completion and at 6-month follow-up. This suggests sustained improvements in overall well-being and reinforces the value of advanced RT modalities. Importantly, the integration of quality-of-life outcomes with economic evaluation provides a more comprehensive assessment of value. While conventional approaches may appear cost-saving, their limited effectiveness and higher uncertainty reduce their overall benefit. In contrast, IMRT and VMAT demonstrate a more favorable balance between costs and outcomes, aligning with the principles of value-based healthcare [20].

The real-world nature of this cohort represents an important strength of the study, particularly in the context of variability in radiotherapy provision and access across European healthcare systems [25,35,36]. At the same time, the findings should be interpreted within the context of a single regional hospital setting, where treatment capacity, case mix, workflow, and access to advanced RT techniques may differ from larger tertiary centers.

The inclusion of multiple cancer types reflects routine oncology practice but introduces clinical heterogeneity. Prognosis, treatment intent, toxicity profile, and expected benefit differ by disease site; therefore, the results should not be interpreted as implying a uniform effect across all malignancies, but rather as providing an exploratory overview across a heterogeneous clinical population [36]. The interpretation of ICER results is not uniform across techniques and subgroups highlighting the importance of cautious, context-specific interpretation.

The present analysis should be interpreted as a short-term evaluation, as the time horizon was limited to treatment completion and a 6-month follow-up. While short-term changes in quality of life are clinically meaningful, they do not capture long-term outcomes such as survival, late toxicity, or sustained functional recovery. This limitation may affect the generalizability of the cost-effectiveness results, as longer-term benefits and costs associated with radiotherapy are not fully reflected. Accordingly, ICER estimates should be interpreted within this short-term framework [21]. A longer-term extrapolation using parametric survival modelling and a state-transition (Markov) framework incorporating long-term costs and utilities would provide a more comprehensive assessment of cost-effectiveness, but this was beyond the scope of the present real-world analysis.

Furthermore, the heterogeneity across cancer types and patient subgroups contributes to variability in cost-effectiveness estimates and limits the generalizability of the findings. This limitation is further compounded by the use of an indirect, literature-based comparator (no “RT”), which introduces additional uncertainty. In addition, several subgroup analyses were based on small sample size, which increases uncertainty and limits the robustness of these estimates. Moreover, the observational design of the study may be subject to confounding and selection bias.

Finally, the use of the Spanish EQ-5D-5L value set, due to the absence of a Greek-specific tariff, may not fully reflect local population preferences. However, this approach is consistent with standard practice in European health economic evaluation studies.

Overall, the findings suggest that simpler techniques such as 2D RT may offer short-term economic advantages but are associated with greater uncertainty and limited clinical benefit. In contrast, IMRT and VMAT provide more consistent cost-effectiveness profiles, improved patient-reported outcomes, and clinically meaningful QALY gains.

## 9. Conclusions

This study provides an integrated clinical and economic evaluation of four radiotherapy (RT) techniques—2D, 3D-CRT, IMRT, and VMAT—among oncology patients treated in a real-world hospital setting. By combining cost-effectiveness analysis with patient-reported quality-of-life outcomes, it offers a comprehensive assessment of the value of contemporary RT modalities.

The findings indicate that although 2D RT demonstrated lower costs and favorable ICER values at low willingness-to-pay (WTP) thresholds, this advantage is primarily driven by its lower costs rather than a meaningful clinical benefit, given its substantially lower QALY gains and higher uncertainty. In contrast, IMRT and VMAT provided greater and more consistent QALYs gains, along with more stable cost-effectiveness profiles across a wider range of WTP thresholds.

Overall, modern radiotherapy techniques, particularly IMRT and VMAT, appear to offer greater value when both economic and patient-centered outcomes are considered, supporting their role in value-based, patient-centered healthcare systems.

Future research should focus on larger, multicenter studies with longer follow-up periods to validate these findings and better capture long-term outcomes, including survival, late toxicity, and patient-reported preferences.

## Figures and Tables

**Table 1:** Average Costs, QALYs, and ICERs by Treatment Technique.

Technique	N	Cost (No RT)	QALYs (No RT)	Cost (RT)	QALYs (RT)	ICER ^a^
2D	5	5560.00	0.352	2787.00	0.1394	13,043.27
3D-CRT	56	6425.00	0.511	5365.88	0.5230	Dominant ^b^
IMRT	152	6143.78	0.388	5317.05	0.3820	135,529.51
VMAT	88	7736.59	0.450	9024.23	0.4936	29,945.12
Total	301	6652.07	0.428	6367.93	0.4368	Dominant ^b^

^a^ ICERs are expressed as €/QALY. ^b^ Dominant indicates a strategy with lower costs and higher effectiveness.

**Table 2:** Average Cost, QALYs, and ICERs per Cancer Type Compared with no RT.

Cancer Type	N	Cost (RT)	QALYs (RT)	Cost (No RT)	QALYs (No RT)	ICER
Head and Neck	45	6686.25	0.310	7800.00	0.360	22,275.00 ^a^
Breast	90	5716.70	0.550	6500.00	0.550	Cost–saving ^b^
Lung	30	5724.25	0.370	2300.00	0.110	13,170.19
Prostate	35	9351.20	0.590	7730.00	0.520	23,160.00
Skin	6	4164.50	0.470	5000	0.350	Dominant ^c^
Endometrial	36	6534.08	0.410	9200	0.500	29,621.33 ^a^
Bone Metastasis	11	3770.91	−0.030 ^d^	5000	0.350	3234.45 ^ad^
Rectal	18	6790.60	0.390	5200	0.320	22,722.86
Pancreas	1	5980.00	0.100	7925	0.280	10,805.56 ^a^
Stomach	9	7272.33	0.450	5400	0.370	23,404.13
Bladder	12	5876.75	0.360	8900	0.460	30,232.50 ^a^
Sarcoma	8	4558.44	0.500	6700	0.360	Dominant ^c^
Total	301	6367.93	0.436	6652	0.428	Dominant ^c^

^a^ Cost-saving but less effective (lower cost and lower QALYs compared with no RT). ^b^ Equal QALYs observed; results reflect a cost-saving strategy without incremental health benefit. ^c^ Dominant indicates lower costs and higher effectiveness. ^d^ Negative QALY values reflect utility estimates below zero, corresponding to health states considered worse than death.

**Table 3:** Distribution Parameters for Cost and QALYs by treatment strategy comparator.

Technique	Cost Mean (€)	Cost SD (€)	QALY Mean	QALY SD	Gamma k	Gamma θ	Beta α	Beta β
Comparator	6652.07	1883.88	0.4287	0.1342	12.4683	533.5187	5.4013	7.1979
2D	2787.00	1518.14	0.1394	0.4448	3.3702	826.9652	0.0325	0.2028
3D-CRT	5365.88	1489.98	0.5230	0.7524	12.9694	413.7343	0.2790	1.7210
IMRT	5317.05	1941.41	0.3820	0.2543	7.5008	708.8649	1.0131	1.6389
VMAT	9024.23	2330.96	0.4956	0.2004	14.9883	602.0868	2.5896	2.6356

**Table 4:** Deterministic sensitivity analysis (±20%) of key BSC parameters and impact on ICER values.

Parameter	Base Case	Lower Bound (−20%)	Upper Bound (+20%)	ICER (€/QALY)
Base-case ICER	–	–	–	16,998.86
Cost Comparator	6652.07	5321.66	7982.49	23,092.46–57,090.19
QALY Comparator	0.4287	0.343	0.5144	7040.70–9351.36

## Data Availability

The data presented in this study are available on request from the corresponding author due to ethical and privacy restrictions related to patient-level clinical data.

## Supplementary Materials

The following supporting information can be downloaded at https://www.mdpi.com/article/10.3390/curroncol33040220/s1, Figure S1: Illustrates the CEAC for the four radiotherapy techniques. At lower WTP threshold, the 2D technique dominates in probability. However, as the WTP increases, IMRT and VMAT show improved cost-effectiveness probability; Figure S2: Cost-Effectiveness Plane A. Scatter plot of the probabilistic sensitivity analysis (10,000 iterations) in the cost-effectiveness plane. Each dot represents a simulated ICER outcome for radiotherapy technique compared with baseline; Figure S3: Cost-Effectiveness Plan B. Shows the cost-effectiveness of the Monte Carlo simulation. The majority of ICER estimates fall below the €30,000/QALY threshold line, indicating high probability of cost-effectiveness, especially for IMRT and VMAT; Table S1: Estimated QALYs and Cost for Best Supportive Care; Table S2: Probability of Cost-Effectiveness by Radiotherapy Technique Across WTP Threshold (€).
