# Supplementary material for: Cost-Effectiveness of Radiotherapy and Its Impact on Patient Quality of Life: A Real-World Cost Utility Analysis in Greece
Source: Curr Oncol. 2026 Apr 16;33(4):220. doi: 10.3390/curroncol33040220 (PMC13114412; doi:10.3390/curroncol33040220)

**Supplementary Materials:** The following supporting information can be downloaded:

**Table S1**: Estimated QALYs and Cost for Best Supportive Care

| Cancer Type | QALY | Cost (€) | Source | Justification |
|---|---|---|---|---|
| Colorectal | 0,32 | 6500 | Uyl-de Groot 2018 doi:10.1186/s13561-018-0197-3 | The QALY and cost estimates are lower than those reported for active treatment (e.g., cetuximab) and are considered representative of BSC. |
| Bladder | 0,28 | 5600 | Porte 2024 doi:10.1371/journal.pone.032548 | Porte et al. (2024) evaluated avelumab; the lower QALY value is considered representative of BSC |
| Non-Small Cell Lung Cancer (NSCLC) | 0,3 | 6000 | Mulvenna 2016 (QUARTZ) doi:10.1016/S0140-6736(16)30825-X | The QUARTZ study (Mulvenna et al., 2016) reported QALY ~0.38 for BSC; the lower value used here is conservative but appropriate. |
| Breast | 0,4 | 5000 | Jalali et.al. 2025 doi: 10.1016/j.cpt.2025.08.06 | Utility for HER2+ disease has been reported as 0.584; the value used here is a conservative estimate with average cost assumptions. |
| Ovarian | 0,5 | 10570 | NICE TA284 (2013) | NICE TA284 does not explicitly report BSC QALY values; however, the selected values are consistent with background estimates used in economic models. |
| Liver | 0,25 | 4200 | Cucchetti 2013 ,doi;10.1016/j.bpg.2013.08.21 | Cucchetti et al. (2013) evaluated treatment strategies rather than BSC; the values used here are derived from secondary sources and consistent with reported estimates. |
| Esophagus | 0,4 | 10600 | Garside et.al. 2007 (HTA) | Values are consistent with HTA-based utility estimates (Garside et al., 2007) and are used as proxy estimates for advanced cancer under BSC. |
| Head &Neck | 0,36 | 8700 | Hirschmann et al., 2018 (economic model). https://doi.org/10.1007/s40273-014-0169-3<br>Schernberg et al., 2019 (Cost / burden) https://doi.org/10.2147/CEOR.S198312 | Economic evaluations in recurrent/metastatic HNSCC report QALY estimates around 0.30–0.40 (e.g., Hirschmann et al., 2018). Cost estimates for advanced disease are consistent with reported healthcare expenditures (Schernberg et al., 2019). The selected values (€8,700) are representative of palliative care under BSC. |
| Pancreas | 0,3 | 8500 | Ljungman et.al. 2013 doi:10.1007/s00268-013-2003-z. | Ljungman et al. (2013) reported QALY ~0.30 and cost ~€8,500 for advanced palliative care; these values are consistent with those used in the table. |
| Prostate | 0,36 | 8800 | Lloyd et al., 2015; doi: https://doi.org/10.1186/s12916-015-0405-5 | Utility values in metastatic castration-resistant prostate cancer (mCRPC) have been reported in the range of ~0.6–0.7 in treated populations (e.g., Lloyd et al., 2015). Lower values are expected in the absence of active treatment; therefore, the selected range (0.34–0.40) is considered representative for patients receiving BSC. |
| Renal | 0,26 | 13200 | Hoyle et.al. 2010 doi: 10.3310/hta14020 | Hoyle et al. (2010) reported QALY values of 0.24–0.27 and costs of approximately €13,200 for BSC in advanced RCC; these values are consistent with those used in the table. |

**Disclaimer:** The QALY and cost estimates presented in this analysis are derived from published literature, health technology assessments, and extrapolated values from comparator arms of clinical trials where explicit best supportive care data were not available. In cases where direct values were missing, weighted or proxy estimations were applied based on consistent methodologies across tumor types. As with all model inputs, variability in real-world clinical and economic parameters may affect generalizability, and thus results should be interpreted within the context of these assumptions.

**Table S2:** Probability of Cost – Effectiveness by Radiotherapy Technique Across WTP Threshold (€).

| WTP | 2D | 3D-CRT | IMRT | VMAT |
|---|---|---|---|---|
| 0 | 94,50% | 70,80% | 70,10% | 21,50% |
| 5000 | 82,60% | 46,10% | 64,40% | 26,40% |
| 10000 | 59,60% | 29,20% | 58,00% | 33,30% |
| 15000 | 40,60% | 21,20% | 53,20% | 38,80% |
| 20000 | 30,20% | 17,70% | 50,50% | 42,90% |
| 25000 | 24,80% | 15,90% | 49,00% | 45,60% |
| 30000 | 21,60% | 14,80% | 47,80% | 48,10% |
| 40000 | 18,20% | 13,80% | 46,10% | 51,50% |
| 50000 | 16,30% | 13,20% | 45,10% | 53,20% |
| 60000 | 15,20% | 12,80% | 44,60% | 54,30% |

**Supplementary Figure S1:** Illustrates the CEAC for the four radiotherapy techniques. At lower WTP threshold, the 2D technique dominates in probability. However, as the WTP increases, IMRT and VMAT show improved cost effectiveness probability.

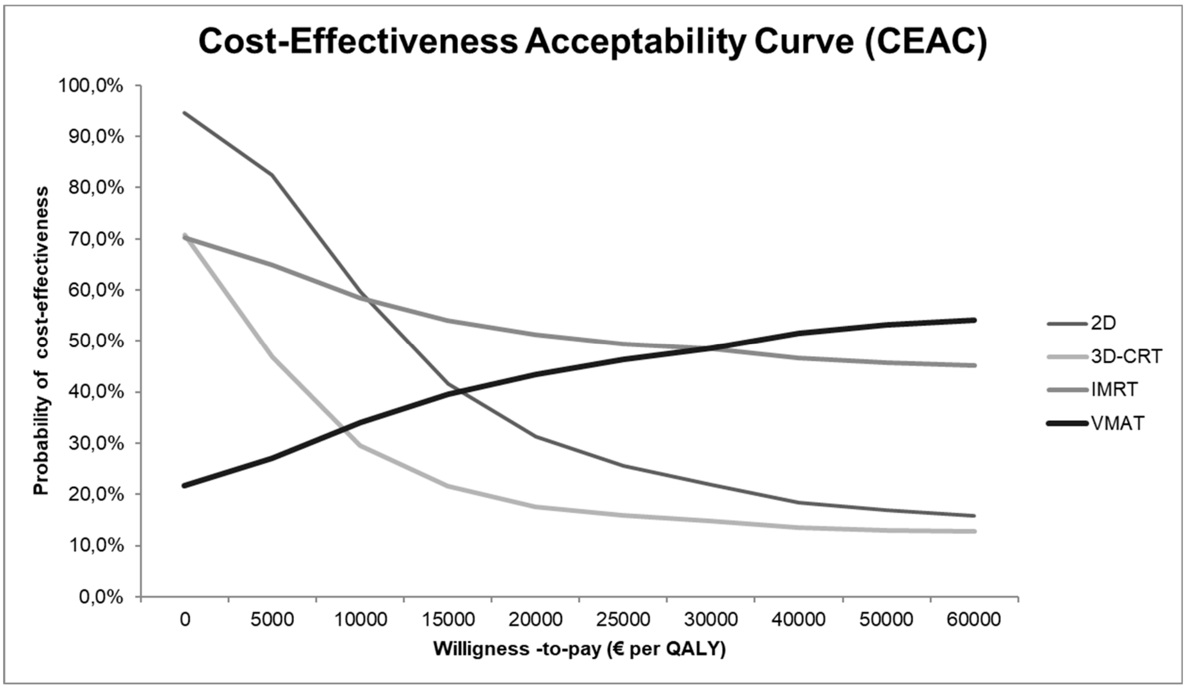

**Supplementary Figure S2:** Cost Effectiveness Plane A. Scatter Plot of the probabilistic sensitivity analysis (10,000 iterations) in the cost-effectiveness plane. Each dot represents a simulated ICER outcome for radiotherapy technique compared with baseline.

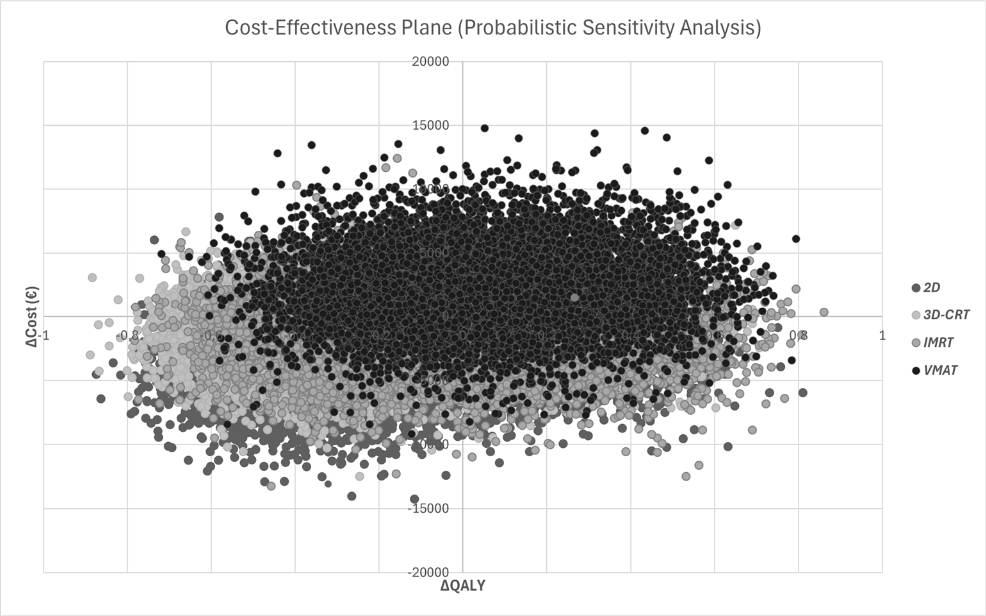


**Supplementary Figure S3:** Cost Effectiveness Plan B. Shows the cost effectiveness of the Monte Carlo simulation. The majority of ICER estimates fall below the € 30.000/QALY threshold line, indicating high probability of cost -effectiveness, especially for IMRT and VMAT.

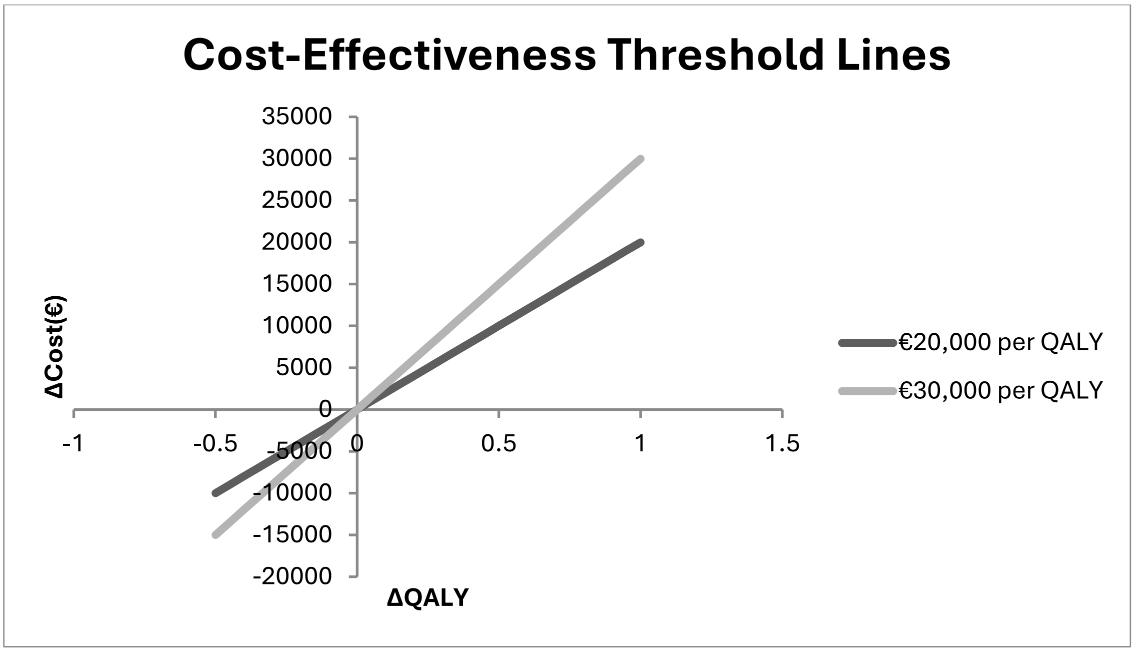

Supplement: Supplementary file 1 [file curroncol-33-00220-s001.zip › curroncol-4229758-supplementary.pdf]
